# Origin and timing of de novo variants implicated in type 2 von Willebrand disease

**DOI:** 10.1111/jcmm.17563

**Published:** 2022-10-13

**Authors:** Ming Chen, Ming‐Ching Shen, Shun‐Ping Chang, Gwo‐Chin Ma, Ying‐Chih Huang, Ching‐Yeh Lin

**Affiliations:** ^1^ Department of Genomic Medicine Changhua Christian Hospital Changhua Taiwan; ^2^ Department of Obstetrics and Gynecology National Taiwan University Hospital Taipei Taiwan; ^3^ Department of Internal Medicine Changhua Christian Hospital Changhua Taiwan; ^4^ Department of Laboratory Medicine and Department of Internal Medicine National Taiwan University Hospital Taipei Taiwan

**Keywords:** de novo variant, type 2 von Willebrand disease, von Willebrand disease

## Abstract

Very few studies have shown the real origin and timing of de novo variants (DNV) implicated in von Willebrand disease (VWD). We investigated four families with type 2 VWD. First, we conducted linkage analysis using single nucleotide variant genotyping to recognize the possible provenance of DNV. Second, we performed amplification refractory mutation system‐quantitative polymerase chain reaction to confirm the real origin of variant (~0% mutant cells) or presence of a genetic mosaic variant (0%–50% mutant cells) in three embryonic germ layer‐derived tissues and sperm cells. Then, three possible timings of DNV were categorized based on the relative likelihood of occurrence according to the number of cell divisions during embryogenesis. Two each with type 2B VWD (proband 1 p.Arg1308Cys, proband 4 p.Arg1306Trp) and type 2A VWD (proband 2 p.Leu1276Arg, proband 3 p.Ser1506Leu) were identified. Variant origins were identified for families 1, 2 and 3 and confirmed to originate from the mother, father and father, respectively. However, the father of family 4 was confirmed to have isolated germline mosaicism with 2.2% mutant sperm cells. Further investigation confirmed the paternal grandfather to be the origin of variant. Thus, we proposed that DNV originating from the two fathers most likely occurred at the single sperm cell, the one originating from the mother occurred at the zygote during the first few cellular divisions; alternatively, in family 4, the DNV most likely occurred at the early postzygotic development in the father. Our findings are essential for understanding genetic pathogenesis and providing accurate genetic counselling.

## 1 INTRODUCTION

von Willebrand disease (VWD) is the most common, inherited bleeding disorder in humans^1^, ^2^ caused by a deficiency or dysfunction of the von Willebrand factor (VWF).^3^ VWF acts as a factor VIII stabilizer and mediator of interaction between platelets and subendothelial tissues.^4^ Gene coding studies have identified the location of *VWF*; it is located on chromosome 12p13.31,^5^, ^6^ consists of 52 exons and encodes a multidomain protein of 2813 amino acids.^7^ VWD is classified into type 1, which is caused by a partial quantitative deficiency of VWF; type 2, which is caused by a qualitative deficiency of VWF and type 3, which is caused by a virtually complete deficiency of VWF.^8^ Type 2 VWD is further classified into types 2A, 2B, 2M and 2N.^8^ Type 1 and 2 VWD, except type 2N, are inherited in an autosomal dominant manner, whereas type 2 N and 3 VWD are inherited in an autosomal recessive manner.^9^

De novo variant (DNV) is a rare phenomenon in VWD.^9^, ^10^ It is defined as a variant observed in a child but not in either parent.^9^, ^11^ Accordingly, family members in whom DNV origins have been recognized are usually free of coagulation alteration and genetic variants associated with the DNV. However, individuals who have mosaic variants that were not detected owing to the use of less sensitive conventional method are not considered as origins of DNV. Previously, there have been several reports on the origin of DNV in VWD,^9^, ^10^, ^12^, ^13^, ^14^, ^15^, ^16^, ^17^, ^18^ but almost no studies have shown the real origin and timing of DNV. Nonetheless, two possible interpretations for DNV occurrence have been described—germline mosaicism in either parent and a DNV in the proband.^9^ Therefore, to understand how DNV occurs, further evaluation of the timing of occurrence of this modification during embryogenesis is required. We previously identified four families with type 2 VWD and DNVs.^19^ In this study, the provenance and timing of DNV during embryogenesis were investigated in the aforementioned four families to understand how this variant occurred. The purposes of this study were to: (1) better recognize the real provenance of DNV, which is distinct from the occurrence of DNV; (2) demonstrate the most likely timing of DNV during embryonic development; (3) understand the genetic pathogenesis, which is critical for the provision of accurate genetic counselling; and (4) provide the parent an explanation for the variant and advice to alleviate the guilt.

## 2 METHODS

### 2.1 Study design

All four patients and their family members provided informed consent, and the study was approved by the Institutional Review Board of Changhua Christian Hospital (IRB approval No.210130). (1.1). First, we conducted linkage analysis using single nucleotide variant (SNV) genotyping to identify the possible provenance of DNV. (1.2) Next, to confirm the real origin of variant with ~0% mutant cells or the presence of somatic and/or germline mosaic variant with 0%–50% mutant cells in various tissues or cells, an improved technique named amplification refractory mutation system‐quantitative polymerase chain reaction (ARMS‐qPCR) was used. If the parents were found to have a mosaic variant with 0%–50% mutant cells in various tissues or cells, their parents (the proband's grandparents) would further be investigated to recognize the real provenance of DNV.(1.3) Finally, the following three possible timing of DNV occurrence during embryonic development were categorized depending on whether the DNV was a complete or a mosaic variant^11^: (1) variant at the single germ cell stage; (2) variant at the zygote stage during the first few cell divisions (both these variant timings would lead to a complete variant in the offspring) and (3) variants occurring early at the postzygotic development stage; this would lead to a mosaic variant.

### 2.2 Diagnosis of type 2 VWD

Coagulation tests, including factor VIII coagulant activity (FVIII:C), von Willebrand factor antigen (VWF:Ag), von Willebrand factor activity (VWF:Act) and VWF multimeric analysis, as well as VWF genetic analysis were conducted as reported previously.^19^

### 2.3 Linkage analysis and identification of the provenance of DNV

**FIGURE 1:**
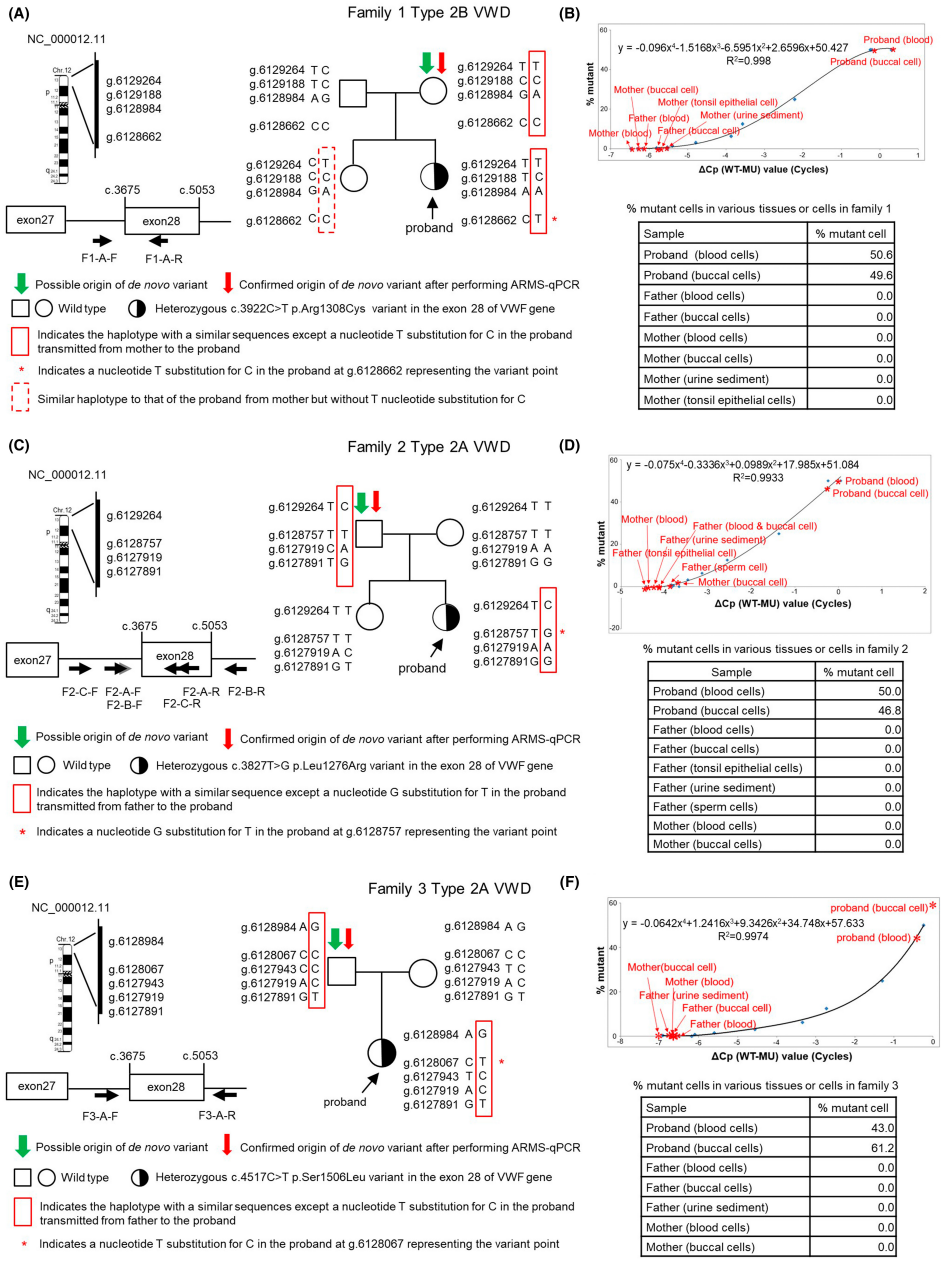
Linkage analysis using single nucleotide variant (SNV)‐genotyping and ARMS‐qPCR to confirm the origin of de novo variant (DNV) in families 1–3. (A), (C) and (E) Linkage analysis using SNV genotyping in family 1, Type 2B VWD; family 2, type 2A VWD; and family 3, type 2A VWD, respectively. The VWD gene location at chromosome 12 (from NCBI data bank, NC_000012.11) are shown at the left side end of each figure with linear genomic sequence marker numbers with a “g” prefix (https://varnomen.hgvs.org/bg‐material/refseq/) selected for linkage analysis. The different arrowhead marks shown at the left side bottom of each figure approximately indicate the size and location of primers designed for linkage analysis: F1, F2 and F3 (families 1, 2 and 3); A, B and C (first, second and third round PCRs); F and R (forward and reverse PCR primers). (B), (D) and (F) Determination of mutant cells (%) using ARMS‐qPCR in various tissues or cells obtained from family members to confirm the origin of DNV (~0% mutant cell) or presence of somatic or germline mosaic variants (0% ~ 50% mutant cell) in families 1, 2 and 3, respectively. ΔCp (WT − MU) represents cycle differences between the qPCR cycle crossing points (Cp) of the wild‐type (WT) and mutant (MU) allele of different synthetic dilutions (X‐axis). Blue points (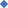) indicate various synthetic dilutions, prepared via a two‐fold serial dilution of MU DNA, using WT DNA, as described in the text (section 2.5). The standard curve in each figure was generated by plotting the ΔCp value (X‐axis) against known mutant percentages of various synthetic dilutions (Y‐axis). The equation for X and Y was then derived. When ΔCp (X) of the test sample after ARMS‐qPCR was known, shown as various red marks, Y (% mutant cell) could be calculated, as shown in each small table.

**FIGURE 2:**
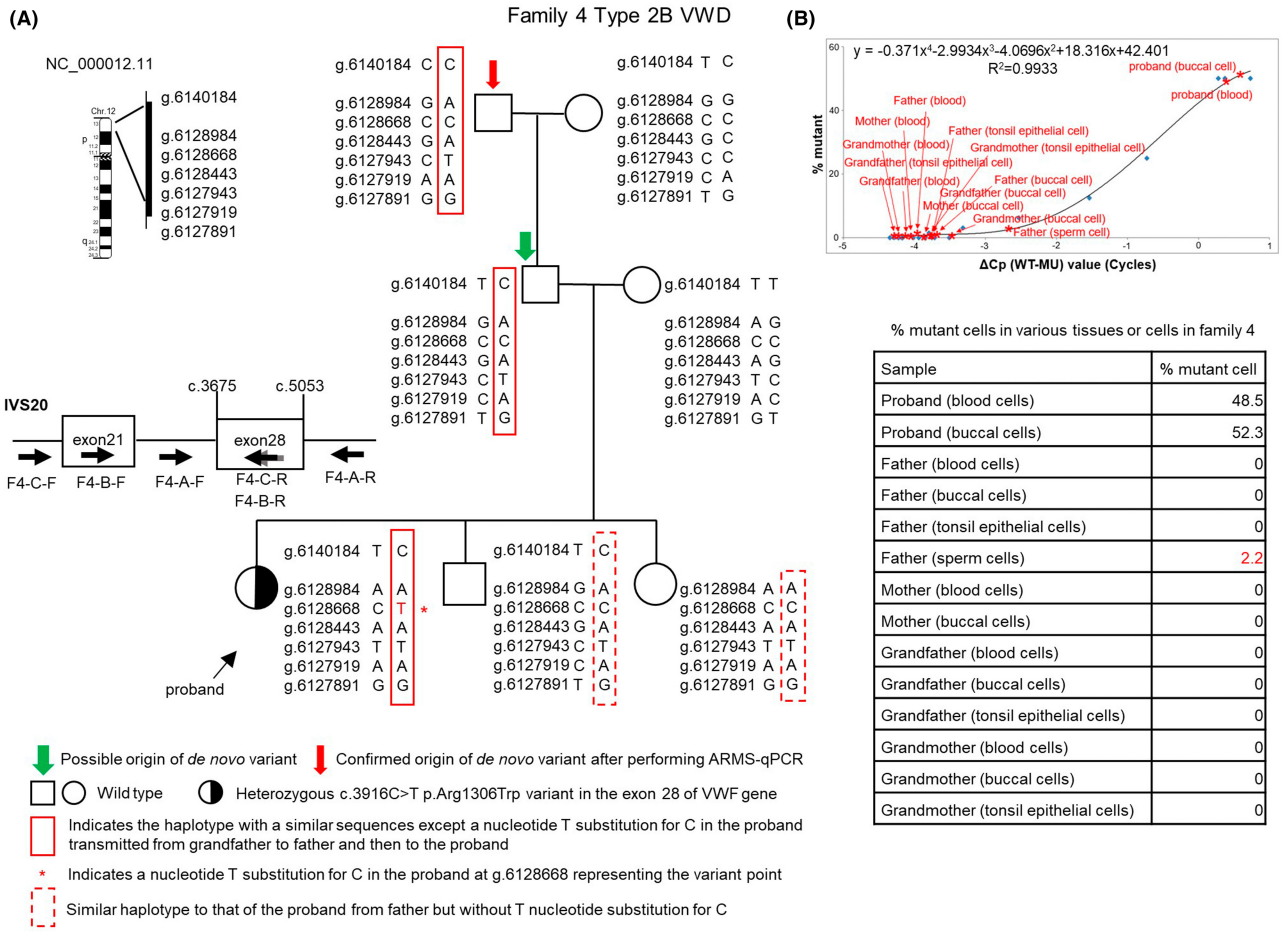
Linkage analysis using single nucleotide variant (SNV) genotyping and ARMS‐qPCR to confirm the origin of de novo variant (DNV) in family 4. (A) Linkage analysis using SNV genotyping in family 4 with type 2B VWD. The location of *VWD* at chromosome 12 (from NCBI data bank, NC_000012.11) is shown at the left side end with linear genomic sequence marker numbers with a g prefix (https://varnomen.hgvs.org/bg‐material/refseq/) selected for linkage analysis. The different marks of arrowheads shown at the left side bottom approximately indicate the size and location of the primers designed for linkage analysis—F4 (families 4); A, B and C (first, second and third round PCRs); and F and R (forward and reverse PCR primers). (B) Determination of %mutant cells via ARMS‐qPCR in various tissues or cells obtained from family members to confirm the real origin of DNV (~0% mutant cell) or the presence of somatic or germline mosaic variant (0%–50% mutant cells) in family 4. ΔCp (WT − MU) represents the differences between the qPCR cycle crossing points (Cp) of the wild‐type (WT) allele and mutant (MU) allele of different synthetic dilutions (X‐axis). Blue points (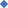) indicate various synthetic dilutions prepared via a two‐fold serial dilution of MU DNA using WT DNA as describe in the text, section 2.5. The standard curve was generated by plotting the ΔCp value (X‐axis) against the known mutant percentage for various synthetic dilutions (Y‐axis). The equation for X and Y was then derived. When ΔCp (X) of the test sample was identified after performing ARMS‐qPCR, was known shown as various red marks, Y (% mutant cell) could be calculated as shown in each small table.

After the VWD variant site was detected using Sanger's method, SNV genotyping^20^, ^21^ was performed using a human *VWF* gene reference sequence (human *VWF* gene‐NCBI database, NC_000012.11; http://www.ncbi.nlm.nih.goo/gene/gene7450) for linkage analysis and identifying the origin of variant among the parents. Initially, primers for genotyping were designed to include the variant site of the proband, then to avoid primer annealing to the VWF pseudogene identified on chromosome 22 (homologous to exons 23–34 of *VWF*, with a 3.1% divergence in sequence),^22^ and finally to ensure that the annealing site did not introduce a mismatch or cause a monoallelic amplification.^23^, ^24^ Subsequently, SNV genotyping was performed repeatedly with different primers to detect more points (SNVs), usually 3–4 points showing differing base pairs between the parents or among the trios to ensure possible discrimination between SNV haplotypes in parents. We observed that almost all the points except the variant site were located in the intron regions. These sites had individual linear genomic reference sequence marker numbers with a “g” prefix, which included not only the exonic but also the intronic nucleotides (https://varnomen.hgvs.org/bg‐material/refseq/) (Figures 1 and 2). Therefore, we selected the SNV haplotype sequence from the trios after they were compared, that is the parent who bore a similar SNV haplotype sequence to the proband, except those variant points with a detected nucleotide substitution that would be assigned to be the possible origin of the variant. When the haplotype sequence is known, each assumed haplotype sequence was further confirmed via polymerase chain reaction (PCR) and sequencing of the indicated genotype. We presented SNV genotyping in family 2 as an example in the Supplemental paragraph.

Subsequently, PCR was performed using a T100TM thermal cycler (Bio‐Rad, Hercules, CA, USA). Each 50‐μL reaction mixture consisted of 100 ng of genomic DNA, 0.2 μM of each primer and 2X SapphireAmp® Fast PCR Master Mix (Takara Bio Inc). Furthermore, PCR conditions were as follows: 94°C for 1 min, followed by 40 cycles at 98°C for 5 s and 68°C for 2 min. Then, sequencing of the PCR product was performed using an automated DNA sequencing analyser ABII3130xl (Applied Biosystems), as reported previously.^25^

Polymerase chain reaction primers used in each round to amplify the three different SNV sites of the parents in families 1–4 are described in Table S1 and those used for the final confirmation of the selected SNV haplotype sequences of the designated origin of variant in families 1–4 are described in Table S2.

### 2.4 Collection of tissue or cells from patients or their family members

Buccal cells, blood cells and palatine tonsil epithelial cells/bladder and urethra epithelial cells are the derivatives of ectoderm, mesoderm and endoderm, respectively.^26^, ^27^ DNAs from these tissues or cells and sperm cells, if available, were isolated for genetic analysis and detection of percent mutant cells as described below.

### 2.5 ARMS‐qPCR to confirm the real origin of the variant

First, to avoid the interference of VWF pseudogenes, preamplification was performed based on the previously published primers^28^, ^29^ and purification was performed using Agencourt AMPure XP beads (Beckman Coulter, Brea, CA, USA) before executing ARMS‐qPCR. ARMS‐qPCR is an effective customized method that can differentially amplify mutant (MU) and wild‐type (WT) alleles. Furthermore, with an enhanced sensitivity of <1% for mutant cell detection, it can recognize the real provenance of DNV. Therefore, to perform the ARMS‐qPCR, a recently reported approach was adopted.^30^ For type 2B VWD with a c.3916C > T, the p.Arg1306Trp mutant of *VW*F, which is similar to the mutated *VWF* identified in the proband of family 4, is shown as an example in Figure S1A,B. In addition, two sequence‐specific forward primers modified with a mismatch at the penultimate nucleotide position of the variant site to augment the specificity of the reaction were also designed (VWF‐3916 T‐mu: 5′‐CCTTTGTGGTGGACATGATGGAAT‐3′ for the MU allele and VWF‐3916C‐wt: 5′‐ CCTTTGTGGTGGACATGATGGAAC‐3′ for the WT allele). The two forward primers were paired with the same reverse primer VWF‐ARMS‐R57: 5′‐GCATACTTCACCTGGCTGGCAAT‐3′ to produce an equivalent 149 bp. All the primers used are shown in Table S3. The ARMS‐qPCR assays were performed in triplicates on a Light Cycler® 480 Real‐Time PCR System (Roche, Rotkreuz, Switzerland). Each 20‐μl reaction mixture consisted of 30 ng of template DNA, 0.3 μmol/L of each primer and 1X Smart Quant Green Master Mix (Protech Technology Enterprise Co). PCR conditions were as follows: 95°C for 10 min, followed by 45 cycles at 95°C for 10 s and 60°C for 30 s.

A standard curve was generated by plotting the difference between the qPCR cycle crossing point (Cp) of the WT allele and MU allele [i.e. ΔCp (WT − MU) value] of different synthetic dilutions (X‐axis) against the mutant percent of the synthetic dilution (Y‐axis) (Figure S1C). Then, we made synthetic dilutions via a 2‐fold serial MU DNA dilution using the WT DNA. An equation for X and Y was then derived. When ΔCp (X) of the test sample is known, Y (% mutant) can be derived.

Once the parent considered to be the origin of variant shows the presence of mosaic variants in any of the obtained tissues (0%–50% mutant cell), their parents (patient's grandparents) should further be examined through ARMS‐qPCR to identify the real provenance of variant.

### 2.6 Determination of possible timing of DNV

The timing of DNV was defined as the periods during embryogenesis, which were categorized as those mentioned by Dr. Freed et al^11^ described in the section of study design. The variant rate is generally known to be essentially correlated with the number of cell divisions—the higher number of cell divisions resulting in more DNA replications, the higher the variant rate. Throughout the development of an embryo, the highest number of cell divisions occurs during spermatogenesis, the second highest number of cell divisions occurs during embryonic mitosis, and the least number of cell divisions occurs during oogenesis.^31^, ^32^ Other mechanisms producing high variant rates include DNA repair of DNA damage and exposure to mutagens.

The possible timing of DNV would be determined after considering two facts—first, who among the parents was the origin of DNV and second, whether it caused a complete variant or a mosaic variant.

## 3 RESULTS

### 3.1 Coagulation studies and DNV of type 2 VWD in the four understudied families

As shown in Table 1, the probands of family 1, 2, 3 and 4 were young girls aged 10, 7, 8 and 12 years, respectively, at the times of diagnosis. All the probands had decreased levels of FVIII:C, VWF:Ag, VWF:Act and VWF:Act/VWF:Ag ratio <0.7 and showed a reduction of large multimers in multimeric analyses (shown in Figure S2); these findings were consistent with those indicating type 2 VWD.^33^ Ristocetin‐induced platelet aggregation revealed either a poor response in probands 2 and 3 (type 2A) or an enhanced response in probands 1 and 4 (type 2B). In contrast, all four parents showed normal levels of FVIII:C, VWF:Ag and VWF:Act and normal distribution in VWF multimeric analysis. Subsequently, since the proband from family 2 was previously characterized as having type 2M VWD,^19^ we changed the VWD classification from type 2M to 2A using an additional multimeric analysis.

**TABLE 1:** Results of coagulation studies performed for four families with type 2 VWD and DNV

Family	Family member	Age, years	Sex	VWD type	VIII:C, IU/dL		VWF:Ag, IU/dL	VWF:Act, IU/dL	RIPA, %				
									Ristocetin concentration, mg/ml				
									0.3	0.6	1.0	1.2	1.5
1	Proband	10	F	2B	31.0		35.9	4.5	18	48	58	90	91
	Father	38	M	NA	88.0		90.4	71.2	ND	ND	ND	ND	ND
	Mother	38	F	NA	92.5		119.2	120.3	ND	ND	ND	ND	ND
2	Proband	7	F	2A	28.4		26.3	6.0	4	6	7	7	12
	Father	32	M	NA	78.0		76.2	71.0	ND	ND	ND	ND	ND
	Mother	32	F	NA	92.7		74.1	65.8	ND	ND	ND	ND	ND
3	Proband	8	F	2A	53.0		38.5	10.0	5	7	7	7	17
	Father	57	M	NA	115.8		272.2	257.2	ND	ND	ND	ND	ND
	Mother	38	F	NA	80.6		66.0	52.5	ND	ND	ND	ND	ND
4	Proband	12	F	2B	27.0		50.0	7.5	3	75	80	85	85
	Father	36	M	NA	93.0		126.0	89.8	ND	ND	ND	ND	ND
	Mother	32	F	NA	55.0		77.0	61.4	ND	ND	ND	ND	ND
			Normal ranges		58–151	O type Non‐O type	52–122 79–308	40.3–125.9 48.8–163.4					

Abbreviations: DNV, de novo variant; F, female; M, male; NA, not applicable; ND, not determinedl; RIPA, ristocetin‐induced platelet aggregation; VIII:C, factor VIII coagulant activity; VWD, von Willebrand disease; VWF:Act, von Willebrand factor activity; VWF:Ag, von Willebrand factor antigen.

While genetic analysis of *VWF* revealed de novo heterozygous c.3922 C to T, p.Arg1308Cys, c.3827 T to G, p.Leu1276Arg, c.4517 C to T, p.Ser1506Leu and c.3916 C to T, and p.Arg1306Trp variants in the blood and buccal cells of the probands 1, 2, 3 and 4, respectively, WT *VWF* was also identified in the four parents. Except for the four probands, all tissues or cells obtained from family members lacked *VWF* variants. Notably, the sperm cells obtained from the fathers of families 2 and 4 also showed the presence of WT *VWF*.

### 3.2 Linkage analysis to identify the possible provenance of the DNV and ARMS‐qPCR to confirm the real origin of DNV

#### 3.2.1 Investigations in families 1–3

Linkage analysis and ARMS‐qPCR of families 1, 2 and 3 are shown in Figures 1A–F, respectively. Nucleotide substitution DNVs are shown using a red asterisk in a haplotype and similar SNV haplotype sequences transmitted in the family are shown using a red rectangle. The final SNV haplotype sequences of each member of the trios, especially those shown using a red rectangle, were confirmed via PCR. The initial linkage analysis disclosed that the possible origin of the DNV was the mother, father and father of families 1, 2 and 3, respectively.

The results of ARMS‐qPCR analysis, as shown in Figure 1B,D and F, revealed that the percentage of mutant cells was ~50% in the blood and buccal cells of the proband of these three families as expected; in contrast, 0% mutant cells were detected in the following cells: blood cells, buccal cells, urinary sediments consisting of epithelial cells of the bladder and urethra or tonsil epithelial cells obtained from all three pairs of parents as well as sperm cells of the father of family 2. These results highly suggested that the real origin of DNV was the mother, father and father of families 1, 2 and 3, respectively. Therefore, these three parents were considered the confirmed provenance of DNV, although no germinal cells were available for study from the mother of family 1 and father of family 3.

#### 3.2.2 Investigations in family 4

The initial linkage analysis disclosed that the possible origin of the DNV originated from the father in family 4 (Figure 2A). The ARMS‐qPCR analysis results (Figure 2B) revealed that the percentage value of mutant cells was ~50% in blood and buccal cells of the proband as expected, but were 0% in blood cells, buccal cells, urinary nucleated cells, tonsil epithelial cells, obtained from the proband's father. In contrast, the percentage of mutant cells was 2.2% in the father's sperm cells, which was undetected by the less sensitive Sanger method. These results indicated that the father had a germline mosaic variant and therefore was not the real origin of the DNV. However, he was assumed to be the origin of variant by initial linkage analysis. Further linkage analysis indicated that DNV originated from the paternal grandfather (Figure 2A). Similar SNV haplotype sequences, except those of the variant point shown in the red rectangle, were also traced downward from the paternal grandfather to father, proband, her young brother and young sister. Moreover, ARMS‐qPCR showed 0% mutant cell in blood cells, buccal cells and tonsil epithelial cells obtained from paternal grandfather (Figure 2B). These results confirmed that paternal grandfather was the origin of the variant.

### 3.3 Possible timing of DNV occurrence during embryonic development

#### 3.3.1 Categorizations in families 1 and 2

**FIGURE 3:**
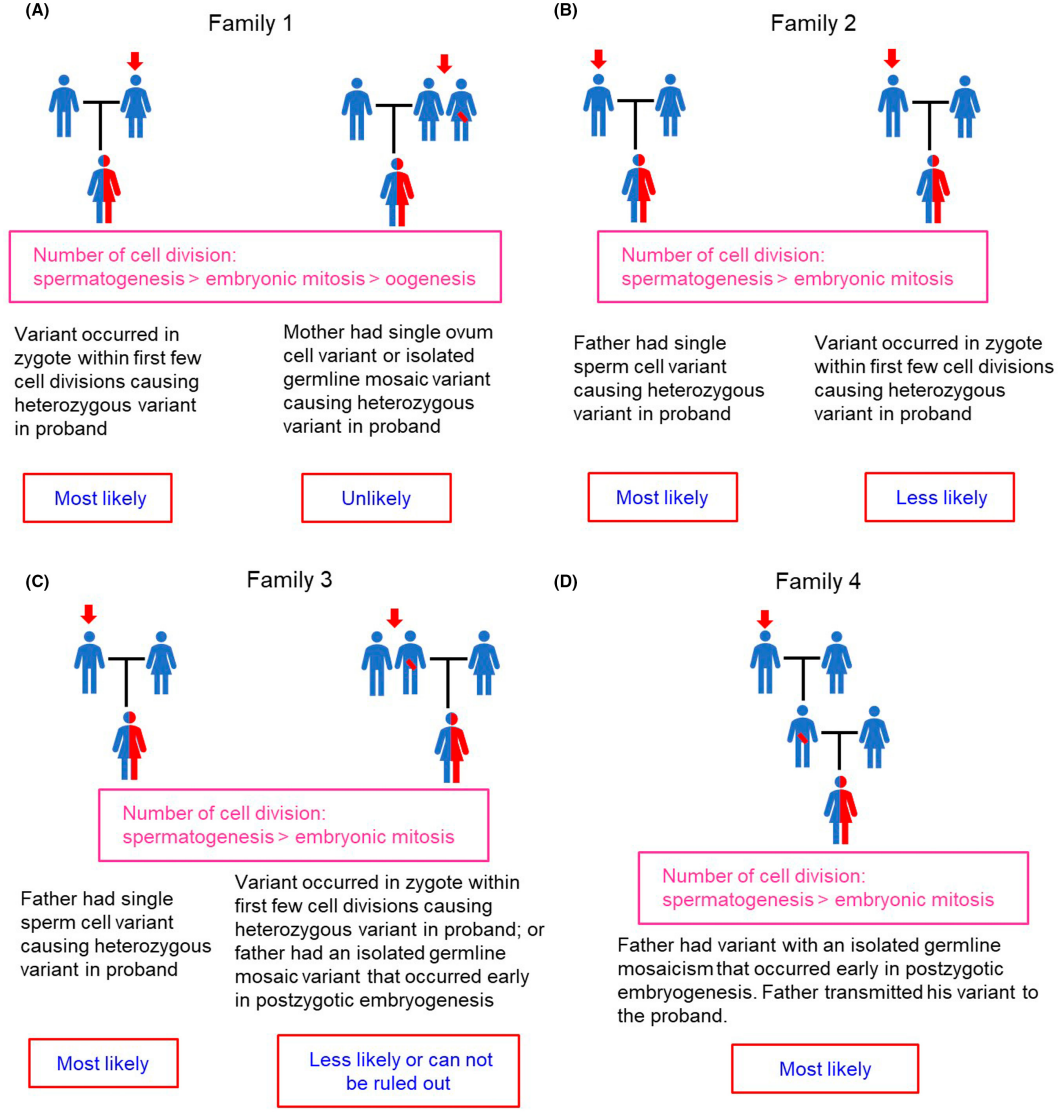
Possible timing of de novo variant occurrence during embryogenesis in family 1 (A), family 2 (B), family 3 (C) and family 4 (D). The most likely possibility is described for each family in comparison with other possibilities based on the relative likelihood of occurrence according to the number of cell divisions and clinical data. The arrow indicates the origin of variant.

The timing of the DNV in families 1 and 2 was evaluated and shown in Figure 3A and B, respectively. We considered that the DNV most likely occurred at the zygote stage during the first few cellular divisions causing heterozygous variant in the proband of family 1; moreover, the father of family 2 had a single sperm cell variant causing heterozygous variant in the proband based on the relative likelihood of occurrence according to the number of cell divisions and clinical data. The other possibility that the mother of family 1 had a single ovum cell variant or isolated germline mosaic variant is unlikely; with reference to family 2, the other possibility that a variant occurred at the zygote stage during the first few cellular divisions causing a heterozygous variant in the proband is less likely.

#### 3.3.2 Categorizations in families 3 and 4

The timing of DNV in families 3 and 4 was investigated and is shown in Figure 3C and D. We observed that the father of family 3 (Figure 3C) most likely had a single sperm cell variant and caused the heterozygous variant in the proband, similar to the event correlated with the father of family 2. However, the other possibility that the DNV occurred at the zygote stage during the first few cell division are less likely, and the possibility that the father had an isolated germline mosaic variant cannot be ruled out as his sperm cells were not available for study. Furthermore, we realized that the father of family 4 (Figure 3D) had a DNV with an isolated germline mosaic variant that most likely occurred early at the postzygotic embryogenesis stage. Therefore, we considered that the father transmitted his variant to the proband.

## 4 DISCUSSION

We considered that the origin of DNV is not the occurrence of DNV, regardless of whether it caused a complete variant or an undetectable mosaic variant. The two events must be distinct from each other so that DNV can be studied. Sanger's variant detection technique is a simple and useful method; however, it has a low sensitivity, requiring a minimum of 5%–20%^34^, ^35^ of mutant cells. Nonetheless, it is still used as a routine method for detecting variants. We assumed that DNV occurred in a family when both the parents were found to have (1) WT *VWF* in the blood and buccal cells, which were easily obtained and (2) normal VWD‐related coagulation data. Moreover, we have performed paternity test in these four families and kinship was confirmed in all families, with the probability of paternity (PP) of 99.99999% (detailed data not shown), as it always cannot be excluded that one father may not be the real parent of the proband. Linkage analysis using intragenic markers^13^, ^16^ or other useful markers^36^ could be employed to recognize the provenance of DNV only when the affected proband had affected offspring. However, this situation was not the case in these four probands. Therefore, we had to employ time‐consuming and laborious SNV genotyping to identify the origin of DNV. Nonetheless, to understand the genetic pathogenesis and offer accurate genetic counselling, the efforts and benefits were worthwhile. Once either parent was identified to be the origin of variant, ARMS‐qPCR, which has a better variant detection sensitivity (<1%), was subsequently used to detect mutant cells originally used for both preimplantation and prenatal genetic diagnoses^37^, ^38^ and confirm the real provenance of DNV (~0% mutant cells), as shown in four families (Figures 1B,D,F and 2B). As previously described, we also collected tissues or cells derived from each of the three embryonic germ layers^26^, ^27^ and sperm cells, if possible, for use as samples for performing ARMS‐qPCR to confirm the real origin of DNV or the presence of mosaic variant. Such extensive tests could hopefully avoid misdiagnosis of the provenance of DNV. Importantly, in family 4, linkage analysis revealed that the proband's young brother and sister (Figure 2A) received the same SNV haplotype sequence from their paternal grandfather (dashed rectangle); however, they did not receive the variant and showed normal coagulation tests (data not shown). These results provided strong evidence to support that the father had an isolated germline mosaicism disorder, which has also been noted in other genetic disorders.^39^, ^40^ Another interesting finding in family 1 was that although the proband's sister received the same SNV haplotype sequence from the mother (dashed rectangle, Figure 1A), she did not carry the same variant. These findings support our notion shown in Figure 3A. Previous studies have reported that variants can occur in a germ cell lineage after the specification of primordial germ cells during early embryonic development,^27^ and these variants remain isolated from somatic cells.^31^, ^41^ These genetic modifications are undetectable in sampled tissues or cells but can be transmitted to the offspring as germline events, similar to those observed in the father of family 4 in our study. In fact, the paternal grandfather was the origin of variant (Figure 3D), although his sperm cells could not be analysed due to their unavailability. However, if the paternal grandfather had an isolated germline variant and no somatic variant, the two rare variational events (one in the grandfather and the other in the father) occurring in one family and causing similar variants will be extremely exceptional.

The three categories of the timing of DNV described by Dr. Freed et al in 2014^11^ and variant rates in correlation with the number of cell division during the development of an embryo are embryogenically justifiable; however, it is impossible to look into an embryo and know exactly the timing of DNV. Therefore, we determined the most likely time during which DNV occurred. All the three categories of DNV timing were observed in this study. Among the four families studied, although complete DNV was found to originate from two males (two fathers), the sample size was small. These findings were consistent with those of previous investigations that showed that major disease‐causing variants with concerned nucleotide substitutions originated from the paternal germline using parent–offspring trios studies.^31^, ^42^ The explanation for this is that a sperm cell undergoes much more germline cell divisions than an egg.^32^, ^43^ Spermatogonial stem cells divide via mitosis approximately every 16 days throughout the male reproductive life, both maintaining their pool and generating differentiated spermatogonia to produce sperm cells. Contrarily, by the time of birth, germ cells in the ovary have already completed their proliferative phase, and only meiosis, which occurs during ovulation, is observed throughout the female reproductive life.^32^ These facts are important data that we rely on to determine the most likely timing of DNV.

In summary, we emphasized that the origin of DNV is distinct from the timing of DNV occurrence and family members designated to be the origin of variant should be free of DNV‐related coagulation alteration and genetic variants. ARMS‐qPCR is an excellent method that fulfilled our study requirements. It is worth noting that DNV was found to originate from three fathers and all the three categories of the timing of DNV were observed in this study, which was evaluated by the relative likelihood of occurrence based on the different number of cell divisions occurring during embryonic development. It is without doubt that the father of family 4 had isolated germline mosaicism. Although no germ cells were available for the mother of family 1 and the father of family 3, other less likely possibilities were not excluded. We believe that our study would contribute toward understanding the genetic pathogenesis and providing accurate genetic counselling.

## AUTHOR CONTRIBUTIONS

**Ming Chen:** Performed ARMS‐qPCR, data analysis, and interpretation. **Ming‐Ching Shen:** Designed the study, collected data, and wrote the article. **Shun‐Ping Chang:** Performed ARMS‐qPCR, data analysis, and interpretation. **Gwo‐Chin Ma:** Performed ARMS‐qPCR, data analysis, and interpretation. **Ying‐Chih Huang:** Performed SNV genotyping for linkage analysis and genetic analysis for diagnosis. **Ching‐Yeh Lin:** Contributed toward the care of patients.

## FUNDING INFORMATION

This study was supported in part by a research fund from the Sanofi Company (TW20002).

## CONFLICTS OF INTEREST

The authors declare that they have no conflicts of interest to disclose.

## PATIENT CONSENT STATEMENT

All patients and their family members provided informed consent.

## Supporting information


Appendix S1

Figure S1

Figure S2

## Data Availability

The identified data generated in this study are available on request to the corresponding author. These data were not publicly available due to ethical restrictions
