# Supplementary material for: Origin and timing of de novo variants implicated in type 2 von Willebrand disease
Source: J Cell Mol Med. 2022 Oct 13;26(21):5403–13. doi: 10.1111/jcmm.17563 (PMC9639050; doi:10.1111/jcmm.17563)
Supplement: Supplementary file 1 — Appendix S1 [file JCMM-26-5403-s003.docx]

**SUPPLEMENTAL PARAGRAPH**

We present SNV genotyping in family 2 as an example. The proband had type 2A VWD with c.3827T > G, p.Leu1276Arg mutant of the VWF gene. The primers for the first round PCR were, forward: VWF-A-F5′-GCCTGACACCGTGGAGACC-3′ and reverse: VWF-A-R5′-CAGGGTGATGCGGGAGGCTTCA-3′ (F2-A-F and F2-A-R shown at the button of Figure 1C). Since only one different SNV site (g.6129264) between the parents was identified by the first round of PCR, the second round of PCR was conducted through a rightward extension (F2-B-F and F2-B-R shown at the button of Figure 1C) with a reading amplicon, including part of the reading amplicon of the first round PCR, and especially the variant point. Primers used for the second round of PCR were, VWF-C-F5′-CGGTCACTTGATTTCACCTGTG-3′ forward and VWF-C-R5′-TGCGCCGCAGCTCTGACGGTCG-3′ reverse. The second round of PCR failed to detect the other distinguishable SNV site. The third round of PCR was performed through a leftward extension (F2-C-F and F2-C-R shown at the button of Figure 1C), using the following primers: VWF-A-F5′-GCCTGACACCGTGGAGACC-3′ forward and VWF-B-R5′-CAGAGGGTGGAATTGGGTGG-3′ reverse. Finally, we identified two SNV sites between the parents. Then, these two SNV sites (g.6127919 and g.6127891) plus the first identified different SNV (g.6129264) and variant (g.6128757) sites were used for discrimination among the trios. Additionally, to confirm that the assumed SNV haplotype sequences were correct, as shown in the red rectangle in Figure 1C, a final PCR was performed. The primers were designed as follows: forward: g.6129264F-T-A-5′CTTGTAAGAAGGCTTGGATTATAGT-3′, g.612964F-C-mt 5′-GATTCTGTGGGAATATGGAAGTTG-3′ and reverse: g.6127891R-T-wt-5′-TGGTAGCGGATCTCTCGGAA-3′, g.6127891R-G-mt-5′-TGGTAGCGGATCTCTCGGAC-3′. PCR results revealed that the SNV haplotype sequences were correct. Accordingly, the father was designated as the origin of the variant (Figure 1C).

**SUPPLEMENTAL FIGURE AND FIGURE LEGEND**

**
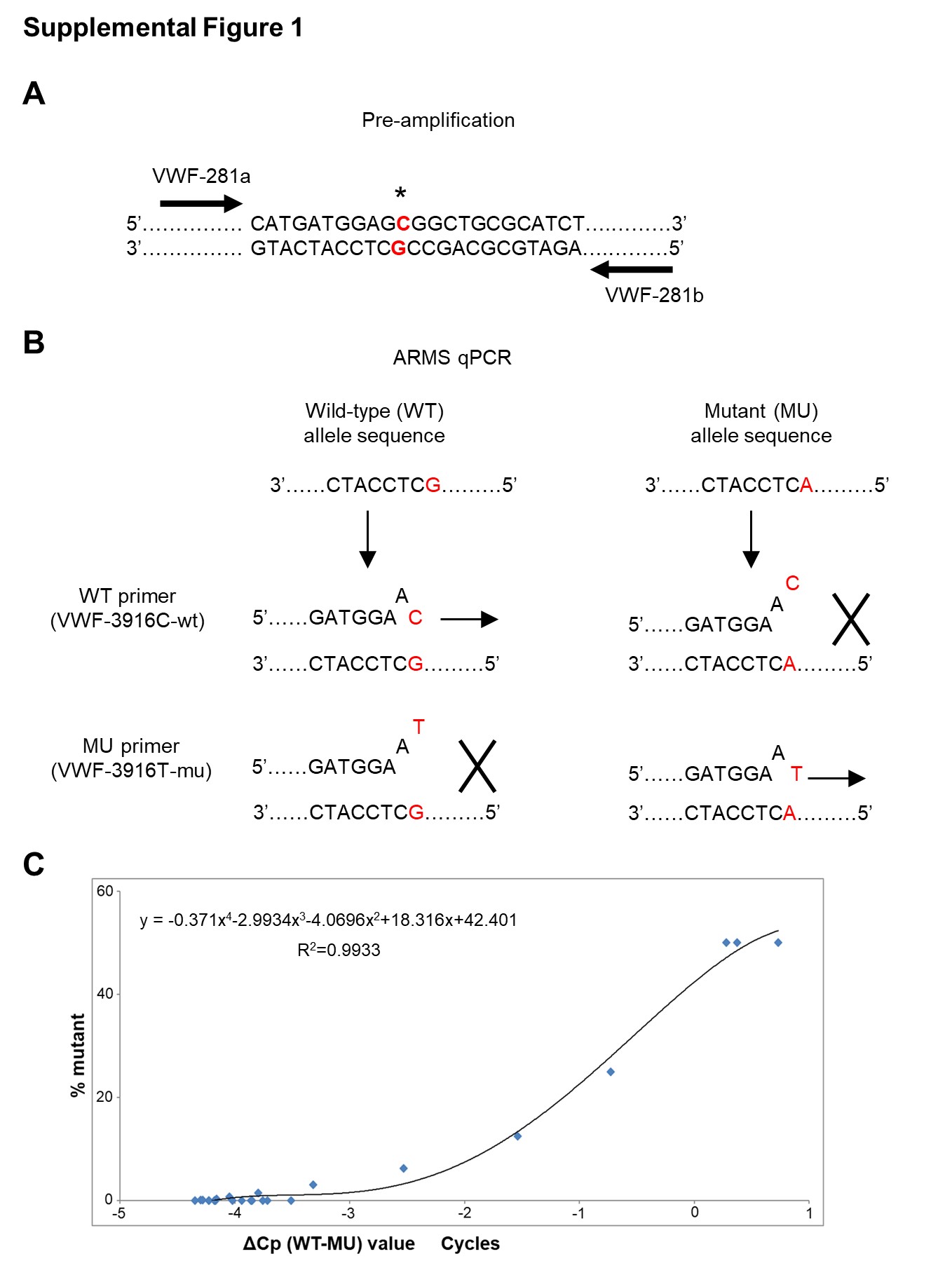
**

**Supplemental Figure 1. Synthetic diagram showing nested ARMS-qPCR for family 4 with the VWF c.3916C > T, p.Arg1306Trp as an example.**

1. The target sequence amplified using specific primers (VWF-281a and VWF-281b) to prevent pseudogenes from interfering with subsequent experiments. Asterisk shows the detected VWF c.3916C > T mutant of the double-stranded DNA. (B) ARMS-qPCR in the case of the type 2B VWD with a heterozygous VWF mutant. The complementary strand (3′–5′ direction) of the mutant (MU) and wild-type (WT) alleles are shown at the top of the figure. Additionally, specific WT and MU primers (shown in the 5’–3’ direction) were custom designed to specifically amplify the WT and MU alleles, respectively. When primers and alleles are paired correctly, subsequent PCR reactions can proceed smoothly. Conversely, PCR was terminated. (C) A standard curve of the family ARMS-qPCR experiment. An equation for X (ΔCp, WT − MU) representing the differences between the qPCR cycle crossing points (Cp) of the WT allele and MU allele of different synthetic dilutions and Y (% mutant, prepared by 2-fold serial dilution of MU DNA by WT DNA shown as blue points) is indicated. When ΔCp (X) of the test sample is known, Y (% mutant cell) can be derived.

**
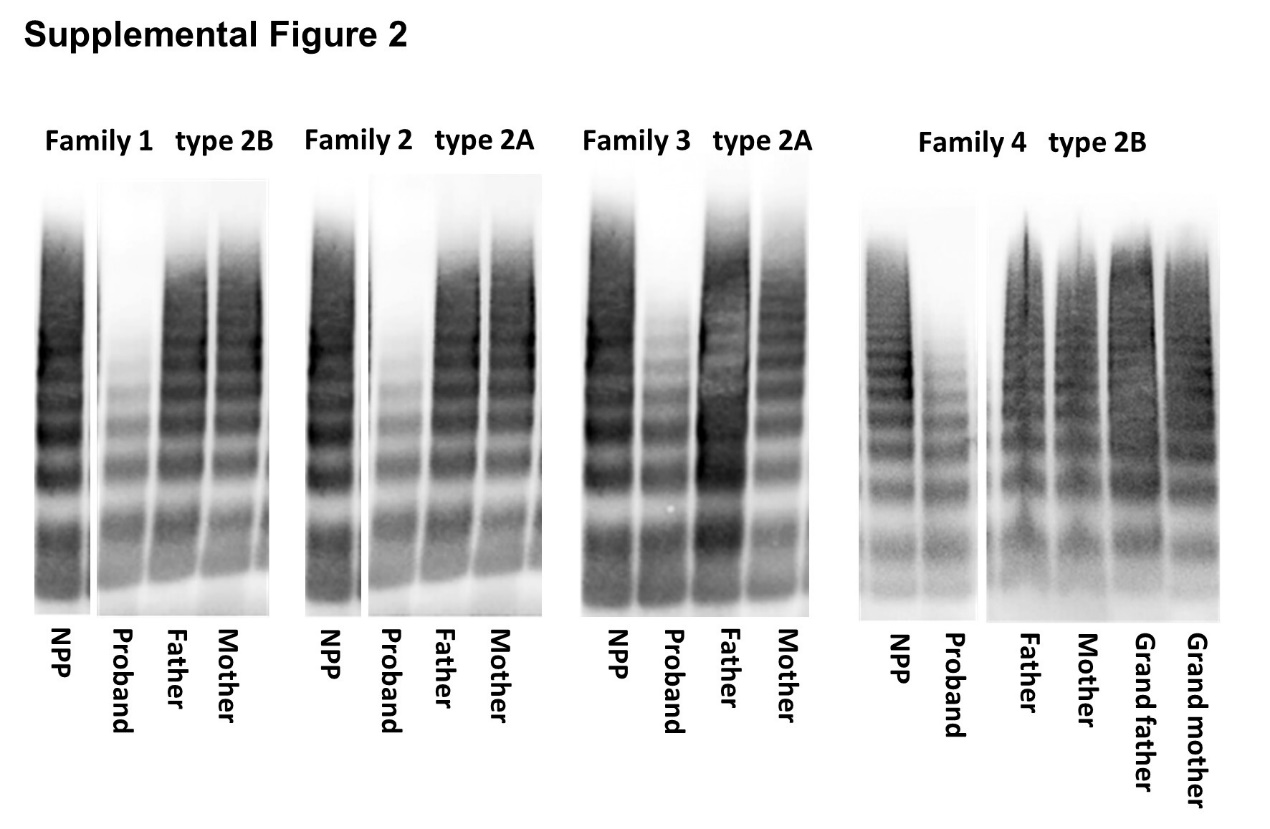
**

**Supplemental Figure 2. Multimeric analysis results revealing plasma von Willebrand factor (VWF).** Multimeric analysis results revealing plasma VWF using 1.2% agarose gel in four families with type 2 VWD and DNVs, according to the method described by previously (Slaughter TF, Parker JK, Greenberg CS. A rapid method for the diagnosis of von Willebrand's disease subtypes by the clinical laboratory. Arch Pathol Lab Med. 1995;119:148-152.)

**SUPPLEMENTAL TABLES**

**Supplemental Table 1. SNV-specific primers used for linkage analysis during SNV genotyping**

| Case No. | Primer^a^ | Primer sequences (5′ to 3′) | Tm, °C | Size (bp) |
| --- | --- | --- | --- | --- |
| Family 1 | F1-A-F | **C**GG**T**CACTTGATTTCACCTGTG | 54.8 |  |
|  | F1-A-R | **T**GCGCCGCAGCTCTGACGGTCG | 64.2 | 1249 |
| Family 2 | F2-A-F | GCCT**G**ACACC**G**TGG**A**GACC | 57.6 |  |
|  | F2-A-R | CAGGG**T**GATGCGG**G**AGGCTTCA | 60.4 | 1119 |
|  | F2-B-F(F2-A-F) | GCCT**G**ACACC**G**TGG**A**GACC | 57.6 |  |
|  | F2-B-R | **CA**GAGGGTGGAATTGGGTGG | 55.9 | 2382 |
|  | F2-C-F | **C**GG**T**CACTTGATTTCACCTGTG | 54.8 |  |
|  | F2-C-R | **T**GCGCCGCAGCTCTGACGGTCG | 64.2 | 1249 |
| Family 3 | F3-A-F | GCCT**G**ACACC**G**TGG**A**GACC | 57.6 |  |
|  | F3-A-R | **CA**GAGGGTGGAATTGGGTGG | 55.9 | 2382 |
| Family 4 | F4-A-F | GCCT**G**ACACC**G**TGG**A**GACC | 57.6 |  |
|  | F4-A-R | **CA**GAGGGTGGAATTGGGTGG | 55.9 | 2382 |
|  | F4-B-F | TTGACCCTGAAGACTG**T**CC**A** | 51.8 |  |
|  | F4-B-R | CAGGG**T**GATGCGG**G**AGGCTTCA | 60.4 | 2731 |
|  | F4-C-F | AGGCCTGTGATGAAGAGATGCCAAT | 57.7 |  |
|  | F4-C-R | **T**GCGCCGCAGCTCTGACGGTCG | 64.2 | 12275 |
| ^a^According to NC_000012.11 | | |  |  |
| Bold fonts nucleotides represent mismatches between von Willebrand factor gene and pseudogene  F1, F2, F3, F4 (family 1, family 2, family 3, and family 4); A, B, C (first, second, and third round PCRs); F, R (forward and reverse PCR primers). | | | | |

**Supplemental Table 2. Primer pairs used to confirm SNV haplotype sequences identified among parent–proband trios (paternal grandfather in family 4)**

| Case No. | Primer^a^ | Primer sequences (5′ to 3′) | Tm, °C | Size, bp |
| --- | --- | --- | --- | --- |
| Family 1 | g.6129264F-T | CTTGTAAGAAGGCTTGGATTATAGT | 49.9 | 646 |
|  | g.6129264F-C | CTTGTAAGAAGGCTTGGATTATAGC | 52.3 |  |
|  | VWF-3922R-C | ACCCACTTCTGGGAGATGTG | 51.0 |  |
|  | VWF-3922R-T | ACCCACTTCTGGGAGATGTA | 48.2 |  |
| Family 2 | g.6129264F-T | CTTGTAAGAAGGCTTGGATTATAGT | 49.9 | 1417 |
|  | g.6129264F-C | CTTGTAAGAAGGCTTGGATTATAGC | 52.3 |  |
|  | g.6127891R-G | TGGTAGCGGATCTCTCGGAC | 54.1 |  |
|  | g.6127891R-T | TGGTAGCGGATCTCTCGGAA | 54.6 |  |
| Family 3 | g.6128984F-A | GATTCTGTGGGAATATGGAAGTTA | 51.1 | 1136 |
|  | g.6128984F-G | GATTCTGTGGGAATATGGAAGTTG | 53.5 |  |
|  | g.6127891R-G | TGGTAGCGGATCTCTCGGAC | 54.1 |  |
|  | g.6127891R-T | TGGTAGCGGATCTCTCGGAA | 54.6 |  |
| Family 4 | g.6140184F-C | TGTTCTTCTGCTACCGTAATGTGTTAGGC | 60.9 | 12293 |
|  | g.6140184F-T | CTGTTCTTCTGCTACCGTAATGTGTTAGGT | 59.5 |  |
|  | g.6127943R-T | GGTCAGGAGTTCGAGACCAGCGTA | 60.5 |  |
|  | g.6127943R-C | GTCAGGAGTTCGAGACCAGCGTG | 59.8 |  |
| ^a^According to GRCh12p13.31 | |  |  |  |

**Supplemental Table 3. Primer pairs used for preamplification and ARMS-qPCR**

| Case No. | Primer^a^ | Primer sequences (5′ to 3′) | Tm, °C | Size, bp |
| --- | --- | --- | --- | --- |
| **Preamplication** | |  |  |  |
| Family 1 | VWF-281a | TGTGGGAATATGGAAGTCATTG | 51.5 |  |
|  | VWF-281b | GTCCGATCCTTCCAGGACGAAC | 58.4 | 940 |
| Family 2 | VWF-281a | TGTGGGAATATGGAAGTCATTG | 51.5 |  |
|  | VWF-281b | GTCCGATCCTTCCAGGACGAAC | 58.4 | 940 |
| Family 3 | VWF-28c | CAAGGCCTTCGTGCTGAGCA | 58.6 |  |
|  | VWF-28d | CAGGATTAGAACCCGAGTCG | 51.3 | 850 |
| Family 4 | VWF-281a | TGTGGGAATATGGAAGTCATTG | 51.5 |  |
|  | VWF-281b | GTCCGATCCTTCCAGGACGAAC | 58.4 | 940 |
| **ARMS-qPCR** | |  |  |  |
| Family 1 | VWF-3922C-wt | GTGGACATGATGGAGCGGCTAC | 58.1 |  |
|  | VWF-3922T-mu | GTGGACATGATGGAGCGGCTAT | 57.6 |  |
|  | VWF-ARMS-R57 | GCATACTTCACCTGGCTGGCAAT | 59.1 | 149 |
| Family 2 | VWF-3827T-wt | CGATTTCTACTGCAGCAGGCTAGT | 56.1 |  |
|  | VWF-3827G-mu | CGATTTCTACTGCAGCAGGCTAGG | 58.4 |  |
|  | VWF-ARMS-R6 | ACTTCAAACTCAGCCTCGGACAG | 56.4 | 81 |
| Family 3 | VWF-4517C-wt | GCGTTCGTCCTGGAAGGACC | 58.1 |  |
|  | VWF-4517T-mu | GCGTTCGTCCTGGAAGGACT | 55.5 |  |
|  | VWF-ARMS-R4 | GCTGAAGGGGTACTCCACAGTCA | 56.9 | 162 |
| Family 4 | VWF-3916C-wt | CCTTTGTGGTGGACATGATGGAAC | 59 |  |
|  | VWF-3916T-mu | CCTTTGTGGTGGACATGATGGAAT | 58.5 |  |
|  | VWF-ARMS-R57 | GCATACTTCACCTGGCTGGCAAT | 59.1 | 149 |
