# Supplementary figures and images for: Origin and timing of de novo variants implicated in type 2 von Willebrand disease
Source: J Cell Mol Med. 2022 Oct 13;26(21):5403–13. doi: 10.1111/jcmm.17563 (PMC9639050; doi:10.1111/jcmm.17563)

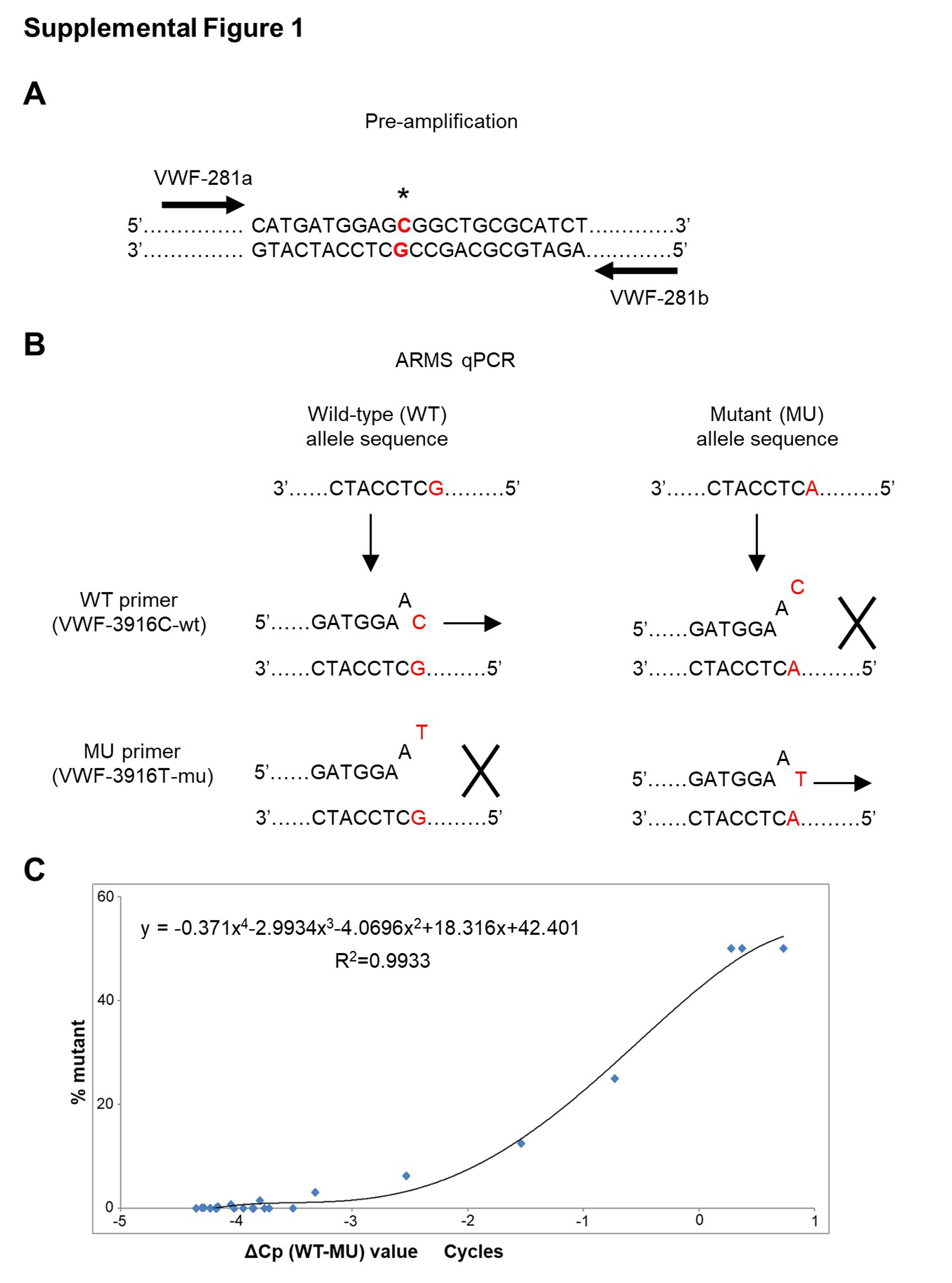

Supplement: Supplementary file 2 — Figure S1 [file JCMM-26-5403-s002.png]

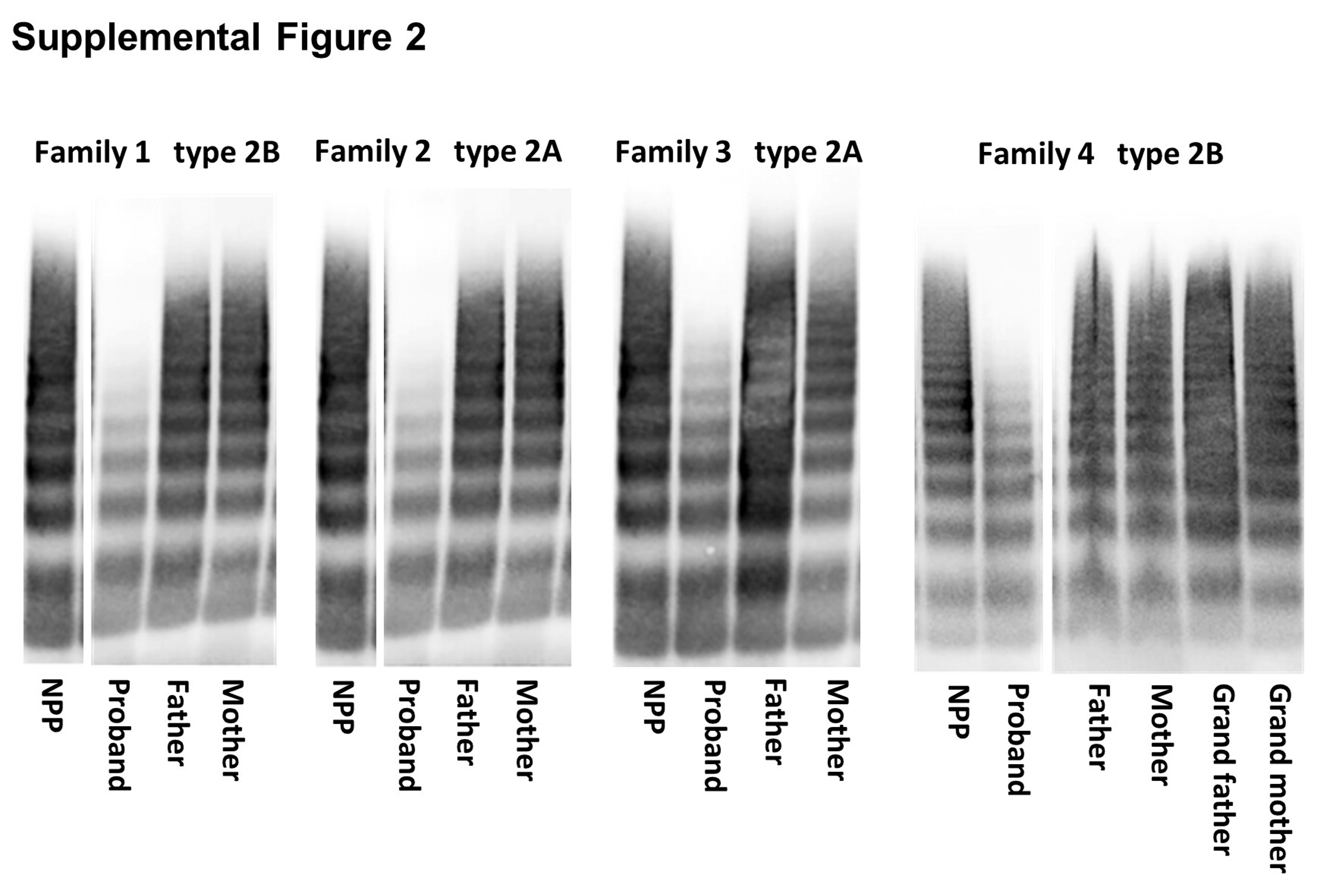

Supplement: Supplementary file 3 — Figure S2 [file JCMM-26-5403-s001.png]
